# Can Internet penetration curb the spread of infectious diseases among regions?—Analysis based on spatial spillover perspective

**DOI:** 10.3389/fpubh.2023.1038198

**Published:** 2023-01-26

**Authors:** Dongsheng Yu, Hui Li, Juanjuan Yu

**Affiliations:** ^1^Zhongnan University of Economics and Law, Wuhan, China; ^2^Wuhan Institute of Technology, Wuhan, Hubei, China

**Keywords:** Internet, infectious diseases, spatial Durbin model, digital gap, COVID-19

## Abstract

Based on the outbreak of COVID-19, this paper empirically studied the impact of internet penetration on the incidence of class A and B infectious diseases among regions in spatial Dubin model, by using health panel data from 31 provinces in China from 2009 to 2018. The findings showed that: (1) The regional spillover effect of incidence of class A and B infectious diseases was significantly positive, and that is most obvious in the central regions. (2) Internet penetration not only has a positive effect on curbing the spread of infectious diseases within the local region but also help to inhibits the proximity spread of infectious diseases in neighborhood, showing the synergistic effect of “neighbor as a partner” in joint prevention and control mechanism. (3) The “digital gap” between regions, urban and rural areas, and user structures had led to significant group differences in the effect of the Internet on suppressing the spread of Class A and B infectious diseases. The findings of this paper provide a reference for understanding the potential role of the Internet in the COVID-19 and also provide policy support for the construction of Internet-based inter-regional “joint prevention and control mechanism” in public health events.

## Introduction

With the further development of Internet technology, the potential of the Internet in public health prevention and control will be further highlighted. Especially since the outbreak of COVID-19, each city or region has promoted “green code” and “health code” based on AI, Internet, big data and other technologies to provide high-precision detection of insensitive and contactless people, avoiding cross infection. The Internet has become a “sharp tool” for precise epidemic prevention. With the development advantages of the Internet and intelligent urban management, we have achieved better anti epidemic effects, which makes us intuitively feel the significance of the rapid development of the Internet in responding to public health emergencies. However, at present, the relevant research mainly focuses on case analysis or qualitative description, lacking relevant empirical analysis and understanding of Chinese characteristics. Over the past 40 years of reform and opening up, with the rapid development of China's economy, the popularity and application of the Internet in China have been greatly improved everywhere, but there are still significant regional differences, urban and rural differences, and user structure differences. How these characteristics affect China's current public health governance remains to be further studied. In addition, infectious diseases are different from common diseases and have the characteristics of “human to human” and “nearby transmission.” The Internet has reduced the contact between people, but it has also increased the connection between people, including information exchange, knowledge sharing, trading, etc., showing a certain “network effect” and “butterfly effect.” So, can the popularization of the Internet curb the regional spread of infectious diseases? If so, is there any difference between regions, urban and rural areas and different users? In the context of the prevention and control of COVID-19, the answers to these questions have important reference value for correctly understanding and grasping prevention and treatment of infectious diseases, so as to propel the sustainable development of Internet.

To explore the answers, based on the social attributes of the Internet and the provincial health panel data of China from 2009 to 2018, this paper uses the Spatial Dubin Model (SDM) to investigate the impact of the Internet penetration level in each province and city on the spread of infectious diseases in the region and nearby areas.

The marginal contributions of this paper can be summarized as below. Firstly, the economic effects of the Internet are widely studied in relevant literature, but few articles have concerned the potential effects of the Internet on public health governance, especially on the prevention and control of infectious diseases in public health. Therefore, this paper is the first to adopt a spatial econometric model, based on provincial health panel data, to examine the impact of the Internet on the incidence of class A and B infectious diseases. Secondly, based on the network effect of the Internet and the “proximity transmission” of infectious diseases, this paper examines the impact of the Internet on the incidence of class A and B infectious diseases within and between regions using the spatial Durbin model, which is helpful for us to understand the importance of “joint prevention and control” between regions in public health events. Thirdly, based on the characteristics of the “digital gap” between different regions, and different users of the Internet in China, this paper provides an in-depth examination of the heterogeneity of the Internet's impact on the spread of infectious diseases, which will help China to tailor its future Internet development and public health governance to local conditions.

The remainder of the analysis is organized as follows: section Literature Review shows the review of related papers. The theoretical basis and typical facts of Internet and the spread of infectious diseases is revealed in Theoretical Basis and Typical Facts. The model construction and variable selection of Internet and the spread of infectious diseases is revealed in section Model and Variables. The data is presented in section Data. The empirical analysis is presented in section Empirical Results. This paper's research is summarized in section Conclusions and Policy Implications. The limitation is presented in section Limitation.

## Literature review

With the development of information technology, China's Internet level has been rapidly improved. Various sharing economies and big data economies supported by the Internet are also changing our production and lifestyle in an all-round way. Research in Internet related fields has also become a hot research topic in the academic community. For example, different scholars discussed the economic effects of Internet development from industrial production (1), industrial upgrading (2, 3), trade and investment (4, 5), enterprise innovation (6, 7), consumption upgrading (8) and other fields.

It is worth noting that some scholars discussed the economic and environmental effects of digitalization. Du et al. (9) discussed the spatial spillover effect and transmission mechanism of digital finance and environmental pollution based on panel data of 30 provinces in China. It is found that with the improvement of digital finance, the inhibition of digital finance on local environmental pollution is gradually enhanced. Peng et al. (10) studied the impact of digitalization on green transformation of enterprises and its mechanism based on text data mining method and the data of Shanghai and Shenzhen A-share listed enterprises from 2011 to 2020. Hao et al. (11) discussed the internal mechanism and linear relationship between digitalization and green economic growth based on panel data from 2013 to 2019, and found that digitalization and green economy growth represent a steady growth trend, and the former as a whole significantly promotes the latter.

Recently, some scholars have studied the impact of the Internet on improving public policy (12), population health (13), and infectious disease control (14) from the field of sociology. The first social function of the Internet is information dissemination, which plays an important role in public health governance. For example, social media sites such as YouTube, Facebook, Twitter, and so on are becoming an important source of information for health protection due to their low cost, fast dissemination, and user interaction in developed countries (15). It is worth noting that after the COVID-19, the diagnosis and death data of COVID-19 epidemic in various regions based on big data let the people all over the country know the real-time situation of the epidemic. Besides, the popularity of the Internet has greatly reduced “face-to-face” contact, which in turn has greatly reduced contact transmission and cross-infection of infectious diseases (16). It is worth noting that after the COVID-19, the Internet has played a significant role in home office, online teaching and online consultation, greatly reducing the risk of epidemic transmission. What's more, the surveillance and prediction function of the Internet can effectively monitor infectious disease and predict its trend (17). It is worth noting that after the COVID-19, we use big data to summarize and analyze, scientifically predict the trend of the epidemic, and provide guarantee for subsequent work arrangements.

The potential of the Internet in public health prevention and control is very obvious, especially for the current COVID-19. However, the current research on infectious diseases is mainly based on case analysis or qualitative description. For example, Zhang et al. (18) collected the information on infectious diseases released by global authoritative institutions with the onset time from 2019 to 2021, and found that influenza, dengue fever and cholera were the top three infectious disease cases. Wang et al. (19) believes that the prevention and control of infectious diseases should be incorporated into the overall framework of national security, the monitoring and early warning system of infectious diseases should be straightened out, and the legal and regulatory system of infectious diseases should be improved. Xie et al. (20) analyzed the trend of population changes and predicted the future incidence of infectious diseases based on ARIMA and SEIR models. It is found that ARIMA model can accurately predict the number of patients in a short period of time, while SEIR model can simulate the changes in the status of various indicators during the period of epidemic changes, and can clearly observe the rising period, inflection point and decline period of infectious diseases, which can more effectively predict the overall trend of infectious diseases. These literatures mostly discuss the types, status quo, prediction, countermeasures and suggestions of infectious diseases, and lack of relevant empirical analysis. There is also little discussion on the relationship between the Internet and infectious diseases. Therefore, this paper conducts an empirical study on the relationship between the two.

## Theoretical basis and typical facts

Unlike ordinary diseases, infectious diseases are characterized by suddenness, urgency, volatility, and widespread. During the COVID-19, we realized the active role of the Internet in responding to public health emergencies, from the fermentation of public opinion and the establishment of the epidemic information platform in the early stage to the timely disclosure of information, online discussion of cases, and publication of treatment plans in the middle stage, as well as the tracking of patients and prevention of the epidemic after the end of the epidemic. Therefore, based on the COVID-19 incident and the theory of infectious disease information, this paper discussed the impact of the Internet on infectious diseases from three stages: source prevention, process control, and post-event management.

Firstly, from the perspective of source prevention: (1) Internet platform monitoring can detect the outbreak of the epidemic from the source and achieve source control. This is widely applied in clinical monitoring and infectious diseases prevention and control abroad. Freifeld (21) found that the Internet plays an important role in the detection and prevention of infectious disease based on Google search volume related to the disease. In addition to public health detection, the Internet can also predict the epidemic development trend from the source and lock the key nodes of the epidemic to prevent the large-scale spread of infectious diseases (22). (2) From the perspective of public health publicity and information dissemination, the application of the Internet can improve the awareness of the public in daily life. Jiang et al. (23) investigated 1,788 residents' attitudes about Internet medical treatment. The study found that more than 60% of residents are willing to accept Internet personalized health assessment and management, and residents have great demand for internet medical and public health publicity. In addition, the acquisition of health information between urban and rural areas in China generally promotes the cultivation of health literacy. Recently, the internet user scales and stickiness have gradually increased. With the change of consumption concept, health information has become an urgent demand and public health literacy would be greatly improved, that helps to reduce the occurrence of public health events and post-event management. (3) From the perspective of contact and transmission of infectious diseases, the popularization of the Internet has greatly reduced face-to-face contact and the cross-infection of infectious diseases. Rapparni et al. (16) found that compared with the high participation of mailing lists, the Internet can be used as an effective tool to improve the prevention of infectious exposure. In the COVID-19, due to the popularity of mobile Internet in China, the public and enterprises are more likely to communicate online, including online shopping, online payment, online sitting, online office, etc., which greatly reduces the frequency of person-to-person contact, thus reducing the contact transmission of infectious diseases.

Secondly, in the stage of process control: (1) From the perspective of information disclosure and knowledge sharing, the Internet platforms such as YouTube and Twitter are rapidly becoming sources of health information in developed countries (15). On the one hand, the widespread use of the Internet has facilitated timely disclosure and information dissemination, reducing the potential transmission caused by information asymmetry. On the other hand, it also promotes the sharing of knowledge and experience in the prevention of infectious diseases, that enhances group identity. The share and spillover of knowledge achieved on the Internet-based platforms have played a positive role in disease transmission and prevention and control (24). (2) From the perspective of treatment experience and location tracking, the Internet has the characteristics of communication and visibility, which can expand the scope of treatment experience exchange and improve the effectiveness of treatment plans. The current emergence of various mobile medical APPs and the improvement of major Internet hospital systems can further integrate medical resources and provide video consultation, co-sitting, and other services (25). On the other hand, based on GIS and big data technology, timely location of confirmed patient, control of the source of infection, dynamic tracking, and such technologies are used to improve the utilization of medical resources (26).

Finally, in terms of post-event management: (1) Application of the Internet allows for the timely detection of infected patients and reduces transmission through nationwide networking and highly precise location. In COVID-19, the health code and green code are widely used in the non-risk areas for tracking prevention and control, which is convenient for screening of enterprises, community employees, and foreigners. (2) From the perspective of basic knowledge awareness, the Internet helps the public to be more concerned about information and knowledge related to infectious diseases. For example, after the SARS outbreak in 2003, Zhang et al. (27) used a group random sampling method to investigate the public awareness of SARS, and the results showed that the ratio of people acquiring knowledge of SARS through media such as promotional leaflets, TV, and radio was over 95%. Therefore, as various new media are more convenient and developed, the public would become aware and knowledge able of preventive measures to the disease, which is helpful to avoid another large-scale outbreak of infectious diseases.

**Figure 1:**
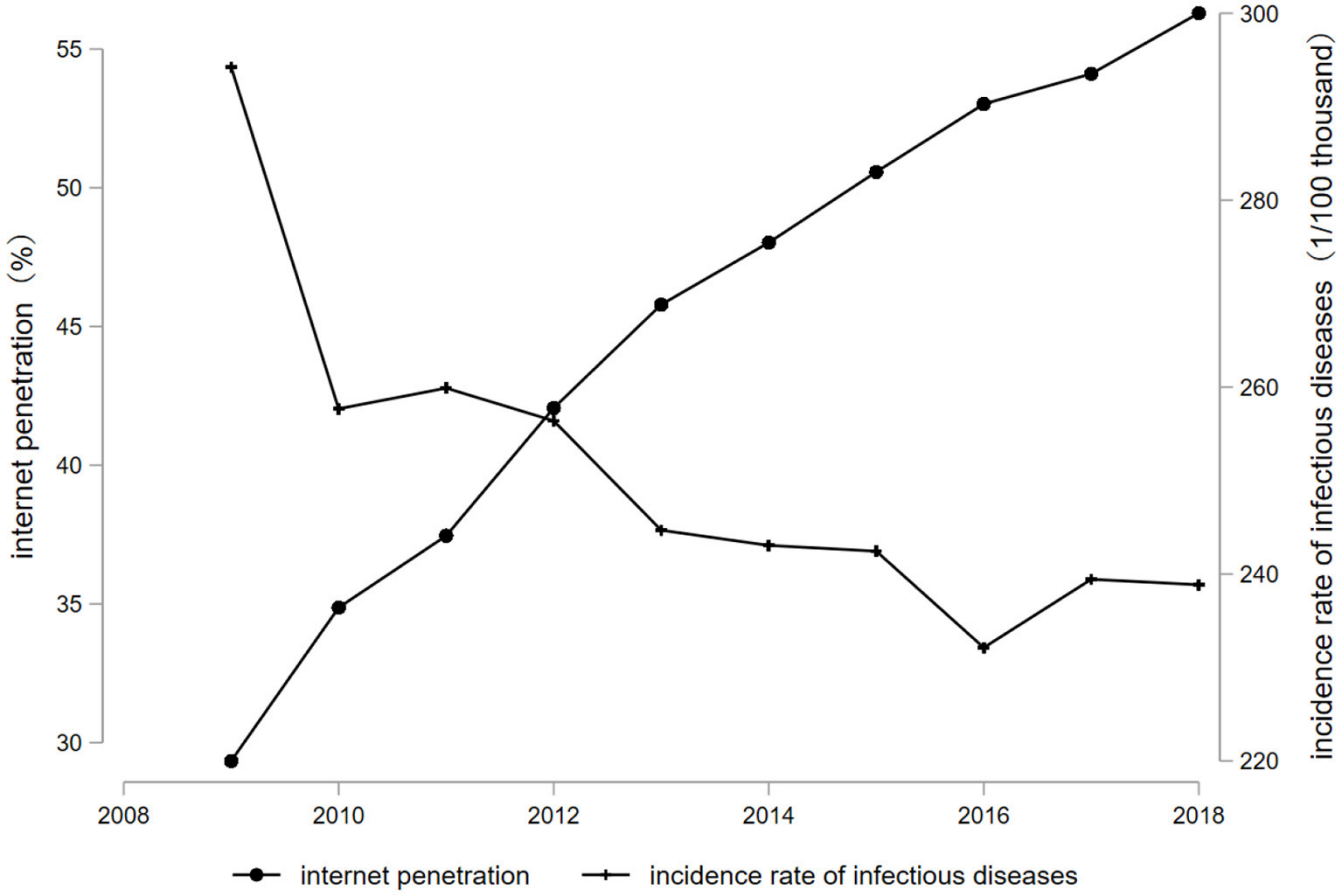
Internet penetration and changes in the incidence of infectious diseases, 2009–2018.

Figure 1 shows the trend of changes in Internet penetration and the incidence of class A and B infectious diseases in each province of China from 2009 to 2018, from which we can visually see that the Internet penetration rate and the incidence of class A and B infectious diseases show an obvious negative relationship. As the level of Internet penetration increases year by year, the incidence of class A and B infectious diseases decreases year by year.

**Figure 2:**
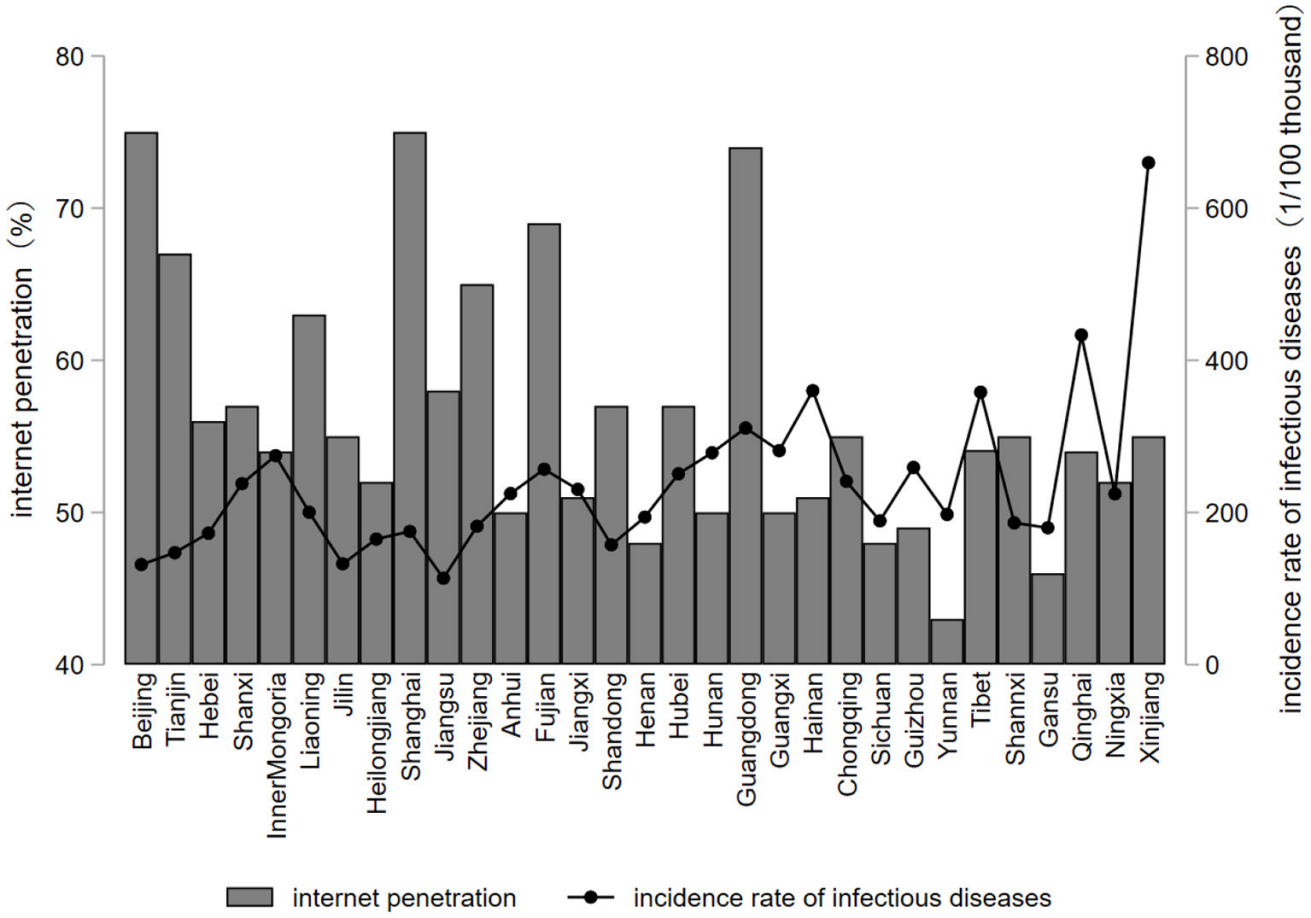
Internet penetration rate and infectious diseases by province, 2018.

Figure 2 shows the level of Internet penetration and the incidence of class A and B infectious diseases by regions in China in 2018, from which we can visually see that the overall development of the Internet penetration level in the eastern region, except for some provinces and cities, is better and generally higher than that in the central and western regions. The top three regions in terms of Internet penetration level are Shanghai, Beijing, and Guangdong, all of which are higher than 70%. On the contrary, the incidence rate of class A and B infectious diseases in the western region is generally higher than that in the eastern region except for some provinces and cities, and the province with the highest incidence rate of class A and B infectious diseases is Xinjiang, followed by Qinghai and Tibet. It can be seen that the provinces and cities with higher levels of Internet penetration have relatively lower incidence rates of class A and B infectious diseases. Landing on the COVID-19, we can see that different cities also show different responses due to the difference in Internet popularity. Among them, Guangdong and Zhejiang in the eastern region took advantage of the development of the Internet to introduce technologies such as big data into urban management, and various online platforms for screening and testing in a short period of time, which played a positive effect on the prevention and control of the epidemic. It was reported that during COVID-19, most of the regions that relied on Internet platforms to open online volunteer hospitals were eastern provinces with a good level of Internet penetration. Therefore, the regional difference of Internet penetration on preventing and controlling infectious diseases is obvious.

**Figure 3:**
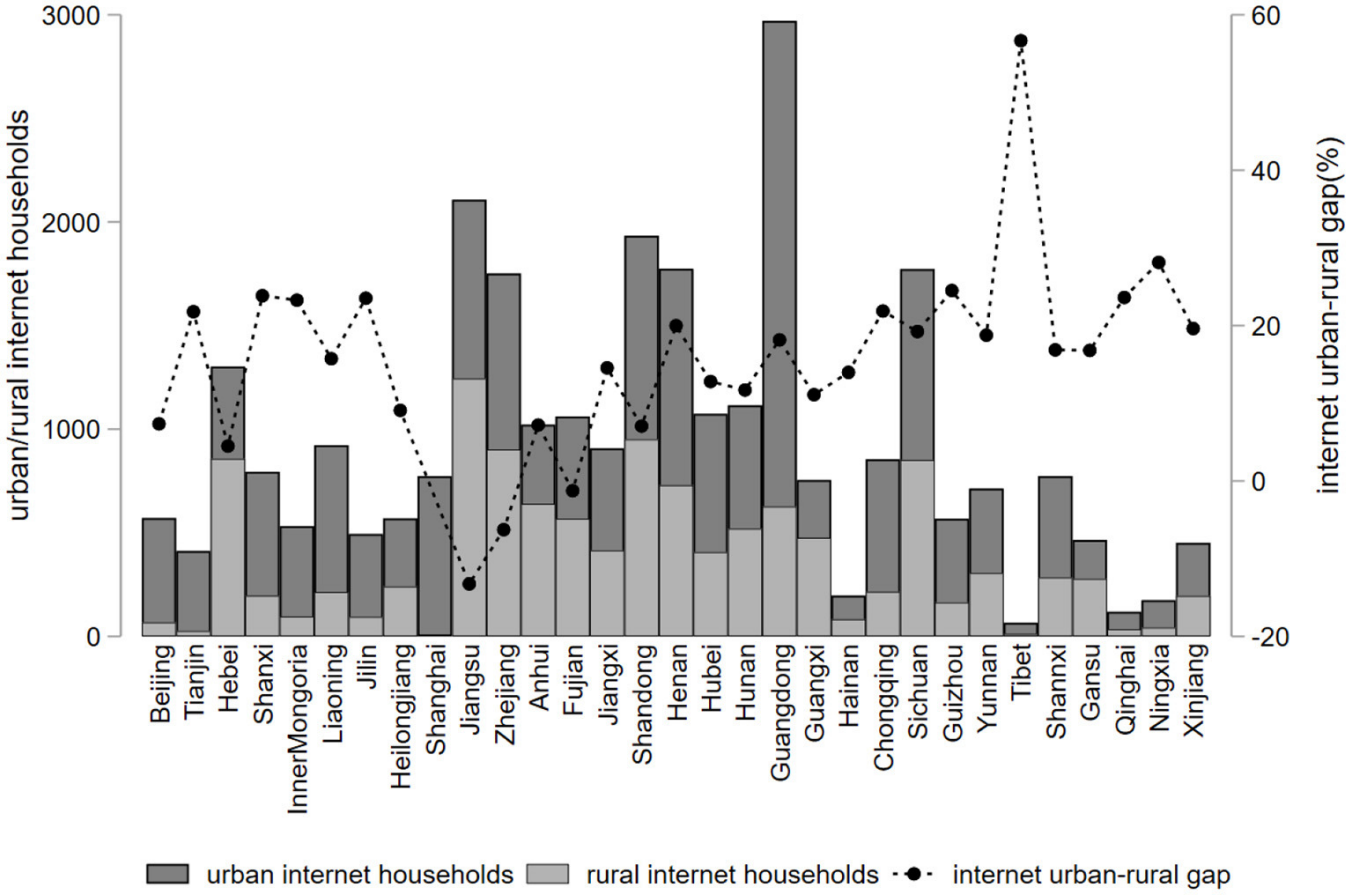
Urban-rural differences on the Internet by province, 2018.

Figure 3 shows the comparison of urban and rural Internet households by region in China in 2018, from which we can visually see that for each region, the number of urban Internet households in the eastern region is generally higher than that in the central and western regions. The top three provinces in the number of urban Internet households are Guangdong, Jiangsu, and Shandong, and the bottom three are Tibet, Ningxia, and Qinghai. For the 31 provinces and cities, the number of urban Internet households is generally greater than the number of rural Internet households, and the difference between urban and rural Internet levels is obvious. Among them, the Internet urban-rural gap in the central and western regions is generally larger than that in the eastern regions. The province with the largest Internet urban-rural gap[Difference between urban and rural Internet levels = urban broadband access users (10,000)/urban population (10,000) - rural broadband access users (10,000)/ rural population (10,000)] is Tibet (56.67%), followed by Ningxia (28.12%) and Guizhou Province (24.05%). According to the latest report of the 44th Statistical Report on Internet Development in China, we found that as of June 2019, the size of China's Internet users reached 854 million, of which 630 million were urban Internet users, accounting for 73.7% of the overall number of Internet users, while the size of rural Internet users was only 225 million, accounting for 26.3% of the overall number of Internet users, which also fully demonstrates the significant different level of urban and rural Internet development in China.

**Figure 4:**
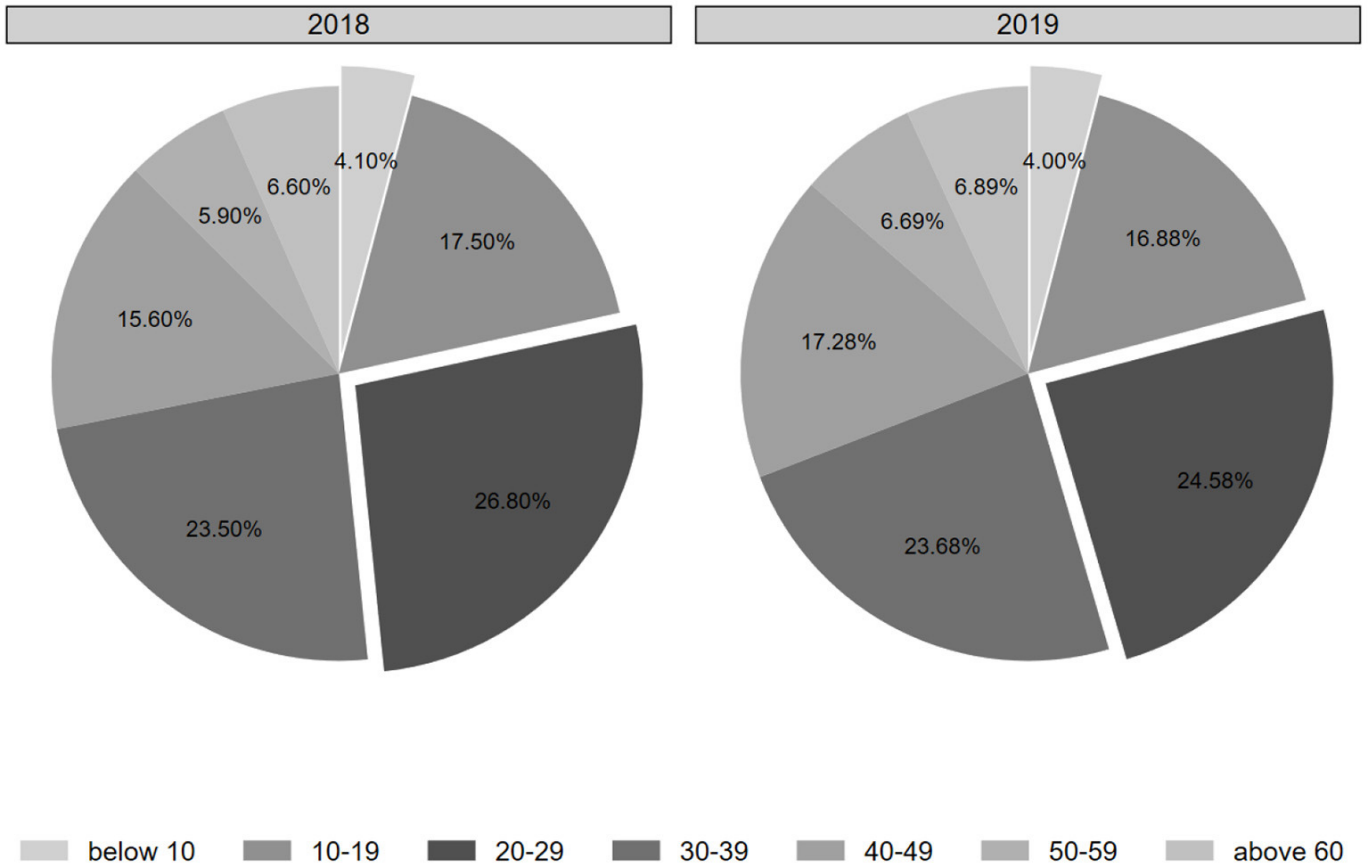
The proportion of Internet users in different age groups.

Figure 4 compares the age structure distribution of China's Internet users in 2018 and 2019. From it, we can see that in the past 2 years, there are obvious differences in different age groups of Internet users, with the highest percentage of Internet users aged 20–29, and the lower percentage of Internet users aged below 10, 50–59, and 60 or above, all accounting for no more than 7%. Specifically, in 2019, the group of Internet users aged 10–49 in China accounted for 82.42% of overall Internet users, but the group of Internet users aged 50 or above only accounted for 13.58% of the overall Internet users, which indicates the “digital age” in the development of the Internet in China among different user groups. When we look at the COVID-19, we can see that due to the low Internet penetration among the elderly, some of them are not sensitive to the reports of the outbreak on the Internet, resulting in a lack of protection knowledge and weak protection awareness of infectious diseases. It was reported that most purchasers of diseases protection products and services online were young people around 25–30 years old, and 66% of young people expected to add new health category consumption.

To sum up, due to regional differences in Internet penetration, urban and rural differences and user use structure differences, there may be some heterogeneity in the impact of the Internet on infectious diseases.

## Model and variables

To examine the impact of Internet penetration on infectious diseases among regions, this paper proposes to use a spatial econometric model for the following reasons: first, different from ordinary diseases, infectious diseases have “person-to-person” and “proximity transmission” characteristics. Therefore, spatial factors such as geographic proximity, transportation convenience, and economic transaction significantly affect the spread of infectious diseases; second, from the perspective of the Internet, there is a certain “network spillover effect” in this kind of interconnection based on information exchange. The use of the Internet has further expanded the boundaries of such information dissemination. Based on this, this paper constructs a spatial econometric model as follows.


(1) $$\begin{array}{l} {\text{disease}}_{\text{it}}=\rho \sum_{j=1}^{n}W_{ij}{\text{disease}}_{\text{j}t}+\alpha {\text{internet}}_{\text{it}}+\beta \sum_{j=1}^{n}W_{ij}{\text{internet}}_{\text{it}}+\\ \omega X_{\text{j}t}+\tau \sum_{j=1}^{n}W_{ij}X_{\text{j}t}+\eta_{i}+\delta_{t}+\mu_{it}\\ \mu_{it}=\text{λ}\sum_{j=1}^{n}W_{ij}\mu_{jt}+\nu_{\text{it}} \end{array}$$

In the above equation (1), *disease* denotes the incidence of infectious diseases of class A and B, and *W*_*ij*_ is the spatial weight matrix. To ensure the reliability of the conclusion, this paper constructed five spatial weight matrix based on the factors of geographical or administrative proximity, geographical distance and economic development. The specific setting process is as follows: First, this paper selects the binary distance matrix (*W*_1_) derived from the macroscopic distance calculated by taking the latitude and longitude of the national capital as the point. The construction principle is to set a certain threshold distance D. Taking the administrative center of a province as the center of a circle, a distance range is drawn with distance D as the radius, and the administrative centers of other regions falling within this distance range are considered to have spatial proximity, and their weights are 1. Conversely, the administrative centers outside this distance range weight 0. Second, this paper constructs a geographic proximity weight matrix (*W*_2_), which is mainly based on whether two provincial units are adjacent on the administrative boundaries; if they are adjacent, their weights take the value of 1, and if not, the weights take the value of 0. Third, drawing on the method of Zhang and Zhu (28), this paper constructs the inverse geographic distance weight matrix (*W*_3_), which is calculated as W_3_ = 1/D_ij_, where D_ij_ denotes the distance between the geographical location in latitude and longitude of province i and province j. Fourth, this paper draws on the method of Elhorst (29) to construct the economic distance weight matrix (W_4_), which is calculated as W_4_ = 1/(|Y_i_-Y_j_|+1), where Y_i_ denotes the annual average value of real GDP per capita in province i and Y_j_ denotes the annual average value of real GDP per capita in province j. Fifth, this paper further constructs the economic geography nested weight matrix (W_5_) by drawing on the method of Shao et al. (30), which is calculated as W_5_ = ϕW_3_+(1-ϕ) W_4_, where the weight ϕ takes the value of 0.5.

Besides, *Internet*_*it*_ denotes the level of Internet penetration of region *i, X*_*it*_ is the control variable in the model, *i* and *j* represent the provincial and municipal cross-sectional units. *η*_*i*_,*δ*_*t*_ represent the individual effect and time effect. μ_*it*_are random disturbance terms. If *ρ* ≠ 0, *β* = 0, and λ = 0, then the model is a spatial lag model (SAR); if *ρ* = 0, *β* = 0, and λ ≠ 0, then the model is a spatial error model (SEM); if *ρ* ≠ 0, *β* = 0, and λ ≠ 0, then the model is a spatial autocorrelation model (SAC); if *ρ* ≠ 0, *β* ≠ 0, and λ = 0, then the model is a spatial Durbin model (SDM); if *ρ* = 0, *β* = 0, and λ = 0, then the model is a general OLS model. For the specific choice of which model, this paper will screen the corresponding model parameters based on the Lratio test, Wald test, and other methods.

Unlike the general regression model, because of the provincial spatial weight matrix set in this paper, the variables among regions may affect each other in addition to their province (called direct effects) and may also have effects on their neighboring regions (called indirect effects). Therefore, this paper draws on Pace & Lesage's (31) interpretation of the spatial model parameters based on the partial derivative matrix to calculate the direct and indirect effects of the independent variables of the spatial Durbin model (SDM) on the dependent variable as follows.


(2) $$\begin{array}{l} \left[\frac{\partial \text{disease}}{\partial X_{1}}...\frac{\partial \text{disease}}{\partial X_{\text{n}}}\right]=\left[\begin{array}{c} \frac{\partial \text{diseas}{\text{e}}_{1}}{\partial X_{1}}...\frac{\partial \text{diseas}{\text{e}}_{1}}{\partial X_{\text{n}}}\\ \cdots \\ \frac{\partial \text{diseas}{\text{e}}_{\text{n}}}{\partial X_{1}}...\frac{\partial \text{diseas}{\text{e}}_{\text{n}}}{\partial X_{\text{n}}} \end{array}\right]\\ \;={\left(I-\rho W\right)}^{-1}\left(\alpha I+\beta w\right) \end{array}$$

In Equation (2), in the partial derivative matrix in the middle of the equation, the mean of the sum of diagonal elements is the direct effect, and the mean of the sum of non-diagonal elements is the indirect effect of the Internet affecting the spread of infectious diseases in adjacent areas, i.e., the spatial spillover effect.

### Explained variables

In this paper, the incidence rate of class A and B infectious diseases is chosen to express the transmission or control of infectious diseases, which is abbreviated as infectious disease transmission in some places in the text. The relevant data was obtained from the Health Statistical Yearbook on the incidence rate of class A and B infectious diseases by province. Previous literature often used indicators such as population mortality (32), life expectancy per capita, and neonatal mortality (33) in measuring the level of public health, but population mortality and final life expectancy are not entirely determined by infectious disease transmission. COVID-19, although never seen in the human category, are now included in the class B infectious diseases. Therefore, to more intuitively reflect the impact of the Internet on the spread of infectious diseases, this paper uses the incidence rate of class A and B legally reported infectious diseases to measure, which to a certain extent can reflect the spread rate and control of infectious diseases, and the selection of indicators is more relevant to the topic and more in line with the current context of the prevention and control of COVID-19.

### The Core explanatory variable

In the theoretical analysis of this paper, we highlight that the Internet influences the spread of infectious diseases by reducing “face-to-face” contact transmission and enhancing “information and knowledge sharing” on the Internet. Therefore, from the perspective of the social attributes of the Internet, this paper selects the indicators related to the level of Internet penetration as the core explanatory variables, which are abbreviated as the Internet in some places in the paper, and adopts the number of Internet broadband access users (10,000) to the total number of households of the resident population (10,000) in the region to reflect the level of Internet penetration. To reflect the urban-rural “digital gap,” this paper uses the number of broadband access users (10,000 households) in Internet cities across the country to compare with the number of rural broadband access users (10,000 households). The greater the ratio, the greater the urban-rural disparity of the Internet in the region. The indicators on the Internet penetration level and the urban-rural gap of the Internet in each province are obtained from the website of the National Bureau of Statistics. Finally, this paper adopts the proportion of Internet users to the total population as a proxy variable for Internet penetration level for robustness testing, where the data on the number of Internet users is obtained from the China Internet Network Information Center.

### Control variables

To enhance the robustness of the regression results, the following control variables are included in this paper concerning relevant literature: **urban population density**, which is measured by the ratio of urban population to total population in each region and reflects a certain extent the regional urbanization process. Intuitively, urbanization will drive “more people” into cities, which may lead to “urban diseases” such as traffic congestion, pollution, and deteriorating public health (34). **Per capita income**, expressed here as GDP per capita, reflects regional economic levels, and Hong and Ahn (35) found that an increase in per capita income is conducive to improving the health of the population and thus better preventing and controlling the spread of infectious diseases. **Cell phone network**, the article focuses on the social attributes of the Internet but ignores the role of the cell phone network, which may overestimate the role of the Internet on the incidence of infectious diseases in class A and B. Therefore, this paper controls for the impact of mobile phone networks in the model, which uses the number of smartphones per capita to measure cell phone networks, with data from the China Internet Network Information Center. **Public health investment**, which expresses the proportion of health expenditure in the total fiscal expenditure of each province and city. **Air quality level**, which is measured by the logarithm of the number of days with air quality reaching and better than level 2 in each province, is directly related to many respiratory diseases, and some pathogens can be freely dispersed in the air for a long time and do long-distance movement (36), thus it exacerbates the spread of infectious diseases.

## Data

All data for the study of this paper were obtained from the 2009–2018 China Statistical Yearbook, China Health Statistical Yearbook provincial and municipal “Statistical Yearbooks” as well as the National Economic and Social Development Statistical Release, China Health and Aging Tracking Survey data, China Internet Network Information Center, National Bureau of Statistics website, Guo Tai Jun An database, and China Economic Network database. The descriptive statistics of each variable indicator in the model are shown in Table 1.

**Table 1:** Descriptive statistics of indicators by city and province.

**Variables**	**Symbol**	**Observed**	**Mean**	**St.d**	**Min**	**Max**
Incidence of Class A and B infectious diseases	disease	310	5.457	0.350	4.630	6.492
Internet penetration level	Internet 1	310	0.456	0.138	0.051	0.78
	Internet 2	310	0.438	0.411	0.098	0.793
Internet urban-rural gap	d-internet	310	3.203	0.530	2.030	9.350
Urban population density	People	310	0.560	0.154	0.007	0.930
GDP per capita	rgdp	310	10.79	0.497	9.303	11.851
Cell phone network	Phone	310	0.45	0.591	0.08	1.30
Public health investment	Expend	310	0.064	0.018	0.027	0.112
Air quality	Air	310	5.628	0.283	3.892	5.903

In addition, the article further examined the spatial correlation between the incidence rate of class A and B infectious diseases and the Internet penetration level in each location using the Moran index, and the results are shown in Table 2. From the results of the global autocorrelation test in Table 3, it is seen that under the geospatial weight matrices W_1_, W_2_, and W_3_, the Moran index is significantly >0 during 2009–2018 for both the incidence rate of class A and B infectious diseases and the Internet penetration level, which indicates that there is a significant positive spatial autocorrelation between the incidence rate of infectious diseases and the Internet penetration level in 31 provinces and cities in China, and the use of the spatial econometric model for regression is appropriate.

**Table 2:** Global autocorrelation test.

**Year**	**Incidence of class A and B infectious diseases**			**Internet penetration level**		

	**W**_1_	**W**_2_	**W**_3_	**W**_1_	**W**_2_	**W**_3_
	**Moran index**			**Moran index**		
2009	0.281^***^	0.212^***^	0.172^**^	0.075^***^	0.0740^***^	0.0900^***^
	(0.000)	(0.007)	(0.026)	(0.007)	(0.001)	(0.000)
2010	0.249^***^	0.114^*^	0.199^**^	0.076^***^	0.0530^***^	0.106^***^
	(0.000)	(0.075)	(0.014)	(0.007)	(0.007)	(0.000)
2011	0.255^***^	0.200^***^	0.133^*^	0.080^***^	0.0750^***^	0.0690^***^
	(0.000)	(0.010)	(0.058)	(0.005)	(0.001)	(0.003)
2012	0.238^***^	0.213^***^	0.164^**^	0.082^***^	0.0750^***^	0.0750^***^
	(0.000)	(0.005)	(0.032)	(0.005)	(0.001)	(0.002)
2013	0.212^***^	0.191^***^	0.171^**^	0.079^***^	0.0630^***^	0.0750^***^
	(0.000)	(0.009)	(0.027)	(0.007)	(0.002)	(0.002)
2014	0.132^***^	0.202^***^	0.206^**^	0.079^***^	0.0680^***^	0.0830^***^
	(0.000)	(0.006)	(0.012)	(0.008)	(0.001)	(0.001)
2015	0.177^***^	0.203^***^	0.161^**^	0.081^***^	0.0650^***^	0.0630^***^
	(0.000)	(0.005)	(0.034)	(0.007)	(0.001)	(0.004)
2016	0.218^***^	0.225^***^	0.18^**^	0.074^**^	0.0730^***^	0.0730^***^
	(0.000)	(0.003)	(0.022)	(0.011)	(0.000)	(0.002)
2017	0.218^***^	0.248^***^	0.175^**^	0.075^**^	0.0780^***^	0.0760^***^
	(0.000)	(0.002)	(0.024)	(0.011)	(0.001)	(0.001)
2018	0.189^***^	0.237^***^	0.169^**^	0.071^**^	0.0700^***^	0.0820^***^
	(0.000)	(0.002)	(0.027)	(0.014)	(0.001)	(0.001)

^*^, ^**^, ^***^denote variables significant at the 10, 5, and 1% levels, respectively, and values in parentheses are *p*-values.

**Table 3:** Spatial econometric model screening results.

	**OLS model**	**RE model**	**FE model**
**General panel screening results**			
LM-SLM	16.662^***^	0.0213	0.0213^**^
LM-SEM	36.701^***^	15.893^***^	15.893^***^
Robust LM-SLM	41.154^***^	0.0317	0.0317^**^
Robust LM-SEM	61.193^***^	15.904^***^	15.904^***^
**Spatial panel model screening results**			
LR-SDM/SEM	20.12^***^	Prob > chi2 = 0.0026	
LR-SDM/SLM	20.53^***^	Prob > chi2 = 0.0022	
Wald-SDM/SEM	18.00^***^	Prob > chi2 = 0.0062	
Wald-SDM/SLM	18.99^***^	Prob > chi2 = 0.0019	
Hausman	19.94^***^	0.0013	
LR-Space Fixed	57.59^***^	Prob > chi2 = 0.0000	
LR-Time Fixed	410.63^***^	Prob > chi2 = 0.0000	

^*^, ^**^, ^***^denote variables significant at the 10, 5, and 1% levels.

## Empirical results

Before conducting the regression analysis, the spatial econometric models need to be screened step by step in the order of “LM test-LR test-Wald test-Hausman test,” and the results are shown in Table 3. First, the LM test is used to determine whether the spatial error model (SEM) or spatial lagged model (SLM) can portray the data better than the model used in the non-spatial panel. The results of the ordinary panel-based tests in Table 3 show that both the ordinary LM test and the robust LM test indicate that the SEM model or SLM model is better at portraying the data than the non-spatial panel model used.

Further screening whether the spatial Durbin model (SDM) is more appropriate than the SLM and SEM models. The results of LR test and Wald test based on the spatial panel model in Table 3 significantly reject the original hypothesis of degradability, which indicates that the use of the spatial Durbin model (SDM) is more suitable. In addition, by the Hausman test and LR test, it is appropriate to choose the spatial Durbin model with temporal and spatial bivariate fixed effects for regression analysis in this paper.

Based on the spatial model screening results in Table 3, the regression results under the constraints of binary distance matrix (W_1_), geographic proximity matrix (W_2_), inverse geographic matrix (W_3_), economic distance matrix (W_4_), and economic geographic nested matrix (W_5_) are reported in this paper using a two-way fixed spatial Durbin model, as shown in Table 4 below. Through Table 4, we can draw the following conclusions.

**Table 4:** Panel regression results of the Durbin model.

	**(1)**	**(2)**	**(3)**	**(4)**	**(5)**

	**W**_1_	**W**_2_	**W**_3_	**W**_4_	**W**_5_
ρ	0.2338^***^	0.2111^***^	0.2530^***^	0.2114^***^	0.2303^***^
	(3.1421)	(3.2640)	(3.8655)	(3.5521)	(3.1165)
Internet 1	−0.0276^**^	−0.0255^*^	−0.0233^*^	−0.0246^***^	−0.0207^**^
	(−2.0131)	(−1.8030)	(−1.7005)	(−3.8033)	(−2.1001)
d-internet	0.0216^**^	0.0224^*^	0.0210	0.0198^**^	0.0222^*^
	(2.0125)	(1.8954)	(1.5011)	(2.1363)	(1.9901)
People	−0.0196^**^	−0.0250^**^	−0.0255^*^	−0.0224^**^	−0.0220^**^
	(−2.2640)	(−2.1320)	(−1.8063)	(−2.0512)	(−2.0774)
rgdp	0.0012	0.0010	0.0011	0.0008	0.0015
	(0.1223)	(0.1127)	(0.1334)	(0.1166)	(0.1662)
Phone	−0.0124^*^	−0.0113^*^	−0.0101	−0.0155^**^	−0.0130^**^
	(-1.9103)	(−1.8245)	(−1.4401)	(−2.0077)	(−2.1033)
Expend	−0.0755^*^	−0.0733	−0.0614^*^	−0.0880^**^	−0.0832^*^
	(−1.8130)	(−1.5223)	(−1.7752)	(−2.1141)	(−1.8805)
Air	−0.0220^*^	−0.0227^**^	−0.0304	−0.0281^**^	−0.0261^*^
	(−1.9136)	(−2.177)	(−1.2244)	(−2.0146)	(−1.8220)
*R*-squared	0.193	0.197	0.224	0.265	0.217
Region effect	Yes	Yes	Yes	Yes	Yes
Time effect	Yes	Yes	Yes	Yes	Yes
Province	30	30	30	30	30
Observations	310	310	310	310	310

^*^, ^**^, ^***^denote variables significant at the 10, 5, and 1% levels, respectively, and values in parentheses are *t*-values.

Firstly, under the five spatial weight matrices, the regression coefficients *ρ* of the spatial lag term in columns (1–5) are all significantly positive, which indicates that there is a significant positive spillover between the incidence of A and B infectious diseases among regions. That is, the incidence of A and B infectious diseases in one province will positively affect the neighboring regions.

Secondly, under the five spatial weight matrices, the regression coefficients of the core explained variable Internet penetration level (internet 1) are significantly negative, which indicates that taking into account the spatial correlation of the variables, the Internet penetration level across the region will reduce the incidence rate of Class A and B infectious diseases in the region. The promotion of Internet popularity can inhibit the spread of Class A and Class B infectious diseases and help to actively prevent and control infectious diseases in the region. This may be because the use of the Internet has significantly reduced the transmission of “person-to-person” contact while ensuring people's basic life and work (15). On the other hand, the Internet can enhance the awareness and protection of the population through information disclosure and knowledge sharing (16), thus inhibiting the spread of infectious diseases. This is also prominent in the COVID-19. In addition, the regression coefficients of the Internet urban-rural gap (*d-internet*) were mostly positive under the five spatial weight matrices, and the vast majority of them passed the significance test, indicating that the widening of the Internet urban-rural gap in each province increases the risk of the development of infectious diseases in class A and B, which is not conducive to the prevention and control of infectious diseases in class A and B. Reducing the “digital gap” between urban and rural areas is of great significance for the “joint prevention and control mechanism.”

Thirdly, we can see the effect of each control variable. The regression coefficients of urban population density (*people*) are significantly negative under the five spatial weight matrices, indicating that the increase in urban population density instead reduces the incidence of infectious diseases, probably because the increase in urban population density is often accompanied by urbanization. And more population density implies the more need for public health and medical resources, which inhibits the spread of diseases to some extent (32). The regression coefficients of GDP per capita (*rgdp*) are insignificant, which indicates that economic development does not necessarily lead to a reduction in the incidence of infectious diseases, probably because the development of public health has market externalities, and an emphasis on economic development may instead deteriorate the local environment and thus make residents fall into the “environmental health poverty trap (37)”. The regression coefficient of mobile phone network (phone) is basically significantly negative, which indicates that the use of mobile phone network can reduce the incidence rate of Class A and B infectious diseases to some extent, similar to the Internet, but its coefficient significance is not as high as that of the Internet on the whole, which may be because there are some online offices, online education, as well as YouTube, Facebook, MySpace, Twitter, SecondLife and other platforms that need more stability High speed networks are connected, and mobile phone networks are not as stable and fast as broadband Internet networks. The regression coefficients of public health input (*expend*) and air quality (*air*) are significantly negative, which indicates that both the increase of public health input and the improvement of air quality are conducive to the reduction of the transmission of class A and B infectious diseases.

To further observe the heterogeneity of regional differences in Internet penetration levels and differences in user structure, we combine the previous analysis framework to regress the samples on two dimensions, “region” and “aging,” respectively, with the binary distance matrix (W_1_) constraint, as shown in Table 5 below. The spatial correlation coefficients *ρ* in columns (1)–(3) are all significant, indicating that the spatial spillover of infectious diseases exists significantly in different regions. Comparatively, the regional spillover effect of infectious diseases is most obvious in the central region, which can be explained by the special geographical location and transportation hub of the central region. From the regression results of *internet* 1, most of the regression coefficients were significantly negative, indicating that the internet has the inhibitory effect on the transmission of class A and B infectious diseases in different regions. However, in terms of regional heterogeneity, the inhibitory effect of the Internet on the spread of infectious diseases is larger than that in the central and western regions. That reason maybe is the level of Internet penetration in the eastern coastal cities is significantly higher than that in the central and western regions.

**Table 5:** Results of heterogeneity analysis.

	**By region**			**By population aging level**	

	**Eastern region**	**Eastern region**	**Western region**	**High–aging region**	**Low–aging region**
	**(1)**	**(2)**	**(3)**	**(4)**	**(5)**
*ρ*	0.2110^***^	0.2856^***^	0.2007^*^	0.2513^***^	0.2520^***^
	(2.4102)	(2.5130)	(1.9055)	(2.8011)	(3.5103)
Internet 1	−0.0270^**^	−0.0210^**^	−0.0199	−0.0203^*^	−0.0281^**^
	(−2.2114)	(−2.0327)	(−1.6037)	(−1.8130)	(−2.1054)
d–internet	0.0130^**^	0.0158^*^	0.0245^*^	0.0246^*^	0.0220^*^
	(2.1012)	(1.8063)	(1.8206)	(1.9905)	(1.8931)
People	−0.0198^*^	−0.0213^**^	−0.0252^**^	−0.0206	−0.0217^**^
	(−1.7063)	(−2.1039)	(−2.0814)	(−1.2160)	(−2.0562)
rgdp	0.0018	0.0015	0.0011	0.0201	0.0155
	(0.1188)	(0.1231)	(0.1110)	(0.1164)	(0.1810)
Phone	−0.0106^**^	−0.0153	−0.0132^*^	−0.0193	−0.0236^**^
	(−2.0513)	(−1.3064)	(−1.9330)	(−1.5103)	(−2.1302)
Expend	−0.0829^*^	−0.0730^*^	−0.0899	−0.0772	−0.0939^*^
	(−1.8230)	(−1.9209)	(−1.5530)	(−1.4434)	(−1.8622)
Air	−0.0225^*^	−0.0208	−0.0133^*^	−0.0117^*^	−0.0206
	(−1.8826)	(−1.0266)	(−1.9016)	(−1.9366)	(−1.6689)
*R*-squared	0.205	0.160	0.177	0.331	0.252
Region effect	Yes	Yes	Yes	Yes	Yes
Time effect	Yes	Yes	Yes	Yes	Yes
Province	12	9	10	17	14
Observations	120	90	100	3,805	3,327

^*^, ^**^, ^***^denote variables significant at the 10, 5, and 1% levels, respectively, and values in parentheses are *t*-values.

In order to further test the difference in the user structure of the Internet, the article divides 30 provinces and cities into “high aging areas” group and “low aging areas” group based on the regional “population aging level” as the distinguishing standard^1^. The regression results are shown in equation 4 and equation 5. By comparing the estimated coefficients, the inhibitory effect of the Internet on infectious diseases in low-aging regions is significantly greater than that in high-aging regions after controlling the effect of other variables. The result may lie in the fact that the weaker learning ability and the lower frequency of using Internet technology among the elderly leads to inadequate dissemination of epidemic information in the high-aging group, which is not helpful for the prevention and control of infectious diseases in the high-aging group. This is also evident in COVID-19. Due to the lack of comprehension of some Internet application, many elderly people do not have adequate information about the epidemic and lack of protective awareness, that led them to be more susceptible to COVID-19 to some extent.

This paper further estimates the direct and indirect effects of the Internet (internet 1) based on the bidirectional fixed effect spatial Dubin model under the binary distance matrix (W_1_), geographical proximity matrix (W_2_) and anti geographic matrix (W_3_). The results are shown in Table 6. Columns (1) is the estimated results for the whole nation of China. The estimated coefficient shows that the direct and indirect effects of the Internet were significantly negative under the regression of the three spatial weight matrices, that indicates the suppressive effect of Internet penetration on infectious diseases not only stays within the region, but also can spill over into adjacent regions, which help to curb the spread of infectious diseases in the neighborhood. This is mainly because Internet penetration can enhance information sharing and knowledge spillover, which would result in a positive spillover on the prevention and government of infectious diseases.

**Table 6:** Spatial spillover effects and regional differences.

		**National**	**Eastern regions**	**Central regions**	**Western regions**

		**(1)**	**(2)**	**(3)**	**(4)**
W_1_	Direct effect	−0.0146^***^	−0.0122^**^	−0.0101^**^	−0.0077
		(−2.9115)	(−2.1103)	(−2.1880)	(−0.9906)
	Indirect effect	−0.0118^*^	−0.0205^**^	−0.0114^*^	−0.0090
		(−1.8066)	(−2.0331)	(−1.91107)	(−0.8776)
	Total effect	−0.0265^**^	−0.0327^***^	−0.0215^*^	−0.0167
		(−2.1103)	(−2.5011)	(−1.8552)	(−0.8830)
W_2_	Direct effect	−0.0133^**^	−0.0150^*^	−0.0160^**^	−0.0112^*^
		(−2.1220)	(−1.7344)	(−2.0530)	(−1.7322)
	Indirect effect	−0.0095^**^	−0.0135^*^	−0.0120	−0.0095
		(−2.0087)	(−1.9001)	(−1.4005)	(−1.1003)
	Total effect	−0.0228^**^	−0.0285^*^	−0.0280^*^	−0.0207
		(−2.0041)	(−1.8870)	(−1.9013)	(−1.4113)
W_3_	Direct effect	−0.0114^***^	−0.0136^*^	−0.0175^*^	−0.0128
		(−3.0554)	(−1.8227)	(−1.9110)	(−1.0006)
	Indirect effect	−0.0099^*^	−0.0103^**^	−0.0155^*^	−0.0108
		(−1.9021)	(−2.1661)	(−1.8733)	(−1.3310)
	Total effect	−0.0213^***^	−0.0239^**^	−0.0330^*^	−0.0236
		(−3.0144)	(−2.0035)	(−1.7998)	(−1.0057)

Note: ^*^, ^**^, ^***^ denote variables significant at the 10, 5, and 1% levels, respectively, and values in parentheses are *t*-values.

Columns (2)–(4) are the estimated results for the eastern, central, and western regions. The comparative analysis revealed that there is significant regional heterogeneity in the spatial spillover effect of the Internet on the incidence of class A and B infectious diseases under the three spatial geographic weight matrices. As we can see, the inhibitory effect of the Internet on infectious diseases is most significant in the eastern region, followed by the central regions. This indicates that for the eastern region, the Internet not only has a positive effect on reducing the transmission of infectious diseases within the region but also inhibits the “proximity transmission” between regions. While the inhibitory effect of the Internet on infectious diseases is insignificant in the western region, that can be explained by the “digital gap” among western regions. The policy implication is that strengthening the Internet penetration in the western region and bridging the “digital gap” between regions will facilitate joint prevention and joint control of infectious diseases and other public health efforts in China.

To further ensure the reliability of the research results, firstly, this paper adopts the number of Internet users as a proportion of the total population (*internet 2*) as a proxy variable for Internet penetration level (*internet 1*) for robustness testing and simultaneously performs spatial spillover effect estimation under five spatial weight matrices as robustness test 1. As shown in Table 7. Secondly, in the spatial spillover effect estimation as well as regional difference analysis, we only consider the effect of geography and do not consider the effect of the economy. Therefore, the spatial spillover estimation results of the economic distance matrix (W_4_) and the economic geographic nested matrix (W_5_) are further reported here as robustness 2, as shown in Table 8. From Table 7, we can see that the regression coefficients of the Internet proxy variables are still significantly negative under the five spatial matrices, and the direction is consistent with the regression results in Table 4, which fully indicates that the Internet can indeed reduce the incidence of class A and B incidence of infectious diseases. From Table 8, we can see that there is also significant regional heterogeneity in the spatial spillover effect of the Internet (*internet 1*) on the incidence of class A and B infectious diseases under the economic distance matrix (W_4_) and the economic geographic nested matrix (W_5_), which is consistent with the direction of the spillover in Table 6, and therefore the spatial spillover estimates of the Internet on infectious diseases are considered to be robust.

**Table 7:** Robustness test 1.

	**(1)**	**(2)**	**(3)**	**(4)**	**(5)**

	**W**_1_	**W**_2_	**W**_3_	**W**_4_	**W**_5_
*ρ*	0.2557^***^	0.2206^**^	0.2513^***^	0.2164^***^	0.2330^***^
	(3.2110)	(3.1981)	(3.8833)	(3.6052)	(3.0174)
Internet 2	−0.0253^*^	−0.0261^**^	−0.0219^*^	−0.0224^**^	−0.0211^**^
	(−1.8155)	(−2.0054)	(−1.7781)	(−2.1044)	(−2.0078)
d-internet	0.0202^**^	0.0213^**^	0.0207^*^	0.0220^**^	0.0241^**^
	(2.1009)	(2.1167)	(1.8906)	(2.0911)	(2.1830)
Control variable	Yes	Yes	Yes	Yes	Yes
Region effect	Yes	Yes	Yes	Yes	Yes
Time effect	Yes	Yes	Yes	Yes	Yes
Province	30	30	30	30	30
Observations	310	310	310	310	310

^*^, ^**^, ^***^ denote variables significant at the 10, 5, and 1% levels, respectively, and values in parentheses are *t*-values.

**Table 8:** Robustness test 1.

		**National**	**Eastern region**	**Central region**	**Western region**

		**(1)**	**(2)**	**(3)**	**(4)**
W_4_	Direct effect	−0.0201^**^	−0.0144^**^	−0.0150^**^	−0.0132
		(−2.1155)	(−2.8555)	(−2.1333)	(−1.0809)
	Indirect effect	−0.0144^*^	−0.0124^**^	−0.0119^*^	−0.0122
		(−1.9033)	(−2.1371)	(−1.8877)	(−1.0195)
	Total effect	−0.0345^*^	−0.0268^***^	−0.0269^**^	−0.0254
		(−1.8226)	(−2.8022)	(−2.0092)	(−0.9895)
W_5_	Direct effect	−0.0157^**^	−0.0133^**^	−0.0166^**^	−0.0118^*^
		(−2.0660)	(−2.0991)	(−2.1990)	(−1.8330)
	Indirect effect	−0.0109^**^	−0.0098^*^	−0.0144^*^	−0.0089
		(−2.2044)	(−2.0365)	(−1.7765)	(−0.9877)
	Total effect	−0.0266^**^	−0.0231^**^	−0.0310^*^	−0.0207
		(−2.0454)	(−2.1110)	(−1.9001)	(−1.2243)

^*^, ^**^, ^***^denote variables significant at the 10, 5, and 1% levels, respectively, and values in parentheses are *t*-values.

## Conclusion and policy implications

In the context of the ongoing COVID-19 outbreak, this paper examined the impact of Internet penetration on the incidence of class A and B infectious diseases within and among regions and observes regional heterogeneity, which provided a reference for understanding the positive role of the Internet in the fight against COVID-19. The conclusions of this study show that: (1) There is a positive spatial correlation between the incidence rates of class A and B infectious diseases among regions, and the characteristic of proximity transmission is most significant in the central region. (2) The increase in Internet penetration not only significantly reduces the incidence rates of class A and B infectious diseases within the local region, but also significantly reduces the incidence rate in the neighboring regions. (3) There is significant heterogeneity in the effect of the Internet on suppressing the spread of Class A and B infectious diseases due to the significant “digital gap” between different regions and user structures.

The policy inspirations of the conclusion in this paper mainly involve the following several aspects:

Firstly, deeply implement the application of Internet technology in the prevention and control of infectious diseases. As the economic effects of the Internet continue to amplify, the potential social effects and health effects of Internet applications need to be further explored and released. COVID-19 has exposed the shortcomings of China's urban public health, but at the same time we have also seen the technological advances in China based on 5G, AI, big data, and other Internet technology in this epidemic prevention. Therefore, in the construction of green and healthy cities, we should further improve the application of Internet technology in public health management, and gradually complete the industrial and urban transformation from “Internet+” to “+Internet” by relying on the public health industry. This includes the use of Internet technology to monitor the source of various infectious diseases, to predict the outbreak of epidemics, and to grasp the best time window for epidemic prevention and control.

Secondly, the Internet-based governmental and social platforms can timely and effectively provide public health knowledge and information, to gradually enhance the intelligence and coordination of public health management and urban management.

Thirdly, the “digital gap” of the Internet challenges the joint prevention and control of public health events. This is reflected in regional heterogeneity. We need to enhance the application of the Internet in western regions and rural areas according to the characteristics of the regions. On the other hand, we need to strengthen the Internet penetration and application among the elderly and improve the awareness of infectious diseases among the elderly to prevent the spread of infectious diseases.

Fourthly, this study shows that the increase in GDP per capita does not significantly reduce the incidence of infectious diseases. The development of public health and wellness has market externalities, and an emphasis on the importance of GDP may instead worsen the local public health and wellness level. Therefore, economic growth should be accompanied by continuous improvement of public services such as medical care, elderly care, and environmental protection that match urban development to better prevent the outbreak of public health events.

## Limitation

This article has two limitations: Firstly, this paper uses the previous data to discuss the relationship between Internet and the spread of infectious diseases. It would be better if it could be combined with the latest COVID-19 data. However, the dimensions of Internet and COVID-19 data are different, so it is impossible to conduct empirical analysis. Therefore, the research of this paper can provide reference for related research in the future. Secondly, there are many factors affecting the spread of infectious diseases. Only some factors can be controlled in this paper, and there is no way to comprehensively consider the impact of other factors on the results in this paper, such as ecological fallacy, solar radiation and so on. These are the focus of our next research.

## Data availability statement

The original contributions presented in the study are included in the article/supplementary material, further inquiries can be directed to the corresponding authors.

## Author contributions

DY: data curation, conceptualization, methodology, and writing—reviewing and editing. HL: data curation and writing—original draft preparation. JY: visualization, writing—original draft preparation, and investigation. All authors contributed to the article and approved the submitted version.

## Footnotes

^1^The specific classification method is to take the average of the aging level of each province during 2009-2018, and then define the provinces above the national average as the group of high aging areas and those below as the group of low aging areas.
